# Mechanistic study of the cause of decreased blood 1,25-Dihydroxyvitamin D in sepsis

**DOI:** 10.1186/s12879-019-4529-7

**Published:** 2019-12-02

**Authors:** Chih-Huang Li, Xiaolei Tang, Samiksha Wasnik, Xiaohua Wang, Jintao Zhang, Yi Xu, Kin-Hing William Lau, H. Bryant Nguyen, David J. Baylink

**Affiliations:** 1 Department of Medicine, Division of Regenerative Medicine, Loma Linda University, Loma Linda, California, USA; 2 Department of Emergency Medicine, Chang-Gung Memorial Hospital, Linkou Medical Center, Taoyuan, Taiwan; 3 Graduate Institute of Clinical Medical Sciences, School of Medicine, Chang-Gung University, Taoyuan, Taiwan; 4 Department of Veterinary Biomedical Sciences, College of Veterinary Medicine, Long Island University, Brookville, NY 11548 USA; 5 Division of Infectious Disease, Jinan Infectious Disease Hospital, Shandong University, Jinan, Shandong China; 6 Institute of Medical and Pharmaceutical Sciences, Zhengzhou University, Zhengzhou, Henan China; 7 Musculoskeletal Disease Center, Jerry L. Pettis Memorial Veterans Affairs Medical Center, Loma Linda, California, USA; 8 Division of Pulmonary, Critical Care, Hyperbaric and Sleep Medicine, Loma Linda University, Loma Linda, California, USA

**Keywords:** Sepsis, 1,25-dihydroxyvitamin D, 25-hydroxyvitamin D 1α-hydroxylase, Insulin-like growth factor 1, Fibroblast growth factor 23, Parathyroid hormone

## Abstract

**Background:**

Vitamin D deficiency, determined by blood levels of 25-hydroxyvitamin D [25(OH) D, i.e. the major vitamin D form in blood], has been shown to associate with all-cause mortalities. We recently demonstrated that blood levels of 1,25-dihydroxyvitamin D [1,25(OH)_2_D, i.e. the active vitamin D] were significantly lower in non-survivors compared to survivors among sepsis patients. Unexpectedly, despite the well documented roles of 1,25(OH)_2_D in multiple biological functions such as regulation of immune responses, stimulation of antimicrobials, and maintenance of barrier function, 1,25(OH)_2_D supplementation failed to improve disease outcomes. These previous findings suggest that, in addition to 1,25(OH)_2_D deficiency, disorders leading to the 1,25(OH)_2_D deficiency also contribute to mortality among sepsis patients. Therefore, this study investigated the mechanisms leading to sepsis-associated 1,25(OH)_2_D deficiency.

**Methods:**

We studied mechanisms known to regulate kidney 25-hydroxylvitamin D 1α-hydroxylase which physiologically catalyzes the conversion of 25(OH) D into 1,25(OH)_2_D. Such mechanisms included parathyroid hormone (PTH), insulin-like growth factor 1 (IGF-1), fibroblast growth factor 23 (FGF-23), and kidney function.

**Results:**

We demonstrated in both human subjects and mice that sepsis-associated 1,25(OH)_2_D deficiency could not be overcome by increased production of PTH which stimulates 1α-hydroxylase. Further studies showed that this failure of PTH to maintain blood 1,25(OH)_2_D levels was associated with decreased blood levels of IGF-1, increased blood levels of FGF-23, and kidney failure. Since the increase in blood levels of FGF-23 is known to associate with kidney failure, we further investigated the mechanisms leading to sepsis-induced decrease in blood levels of IGF-1. Our data showed that blood levels of growth hormone, which stimulates IGF-1 production in liver, were increased but could not overcome the IGF-1 deficiency. Additionally, we found that the inability of growth hormone to restore the IGF-1 deficiency was associated with suppressed expression and signaling of growth hormone receptor in liver.

**Conclusions:**

Because FGF-23 and IGF-1 have multiple biological functions besides their role in regulating kidney 1α-hydroxylase, our data suggest that FGF-23 and IGF-1 are warranted for further investigation as potential agents for the correction of 1,25(OH)_2_D deficiency and for the improvement of survival among sepsis patients.

## Background

In the past decade, there was a rejuvenation of interest in the relationship between vitamin D and infection [1]. As a result, an appreciable amount of data have accumulated to support that vitamin D deficiency as determined by blood 25-hydroxyvitamin D (25[OH]D) levels is associated with all-cause mortalities [2–6]. In our previous studies, we uncovered that blood levels of 1,25-dihydroxyvitamin D (or 1,25[OH]_2_D, i.e. the active vitamin D metabolite) were significantly lower in non-survivor sepsis patients when compared to survivor sepsis patients and hence an independent predictor of sepsis mortality [7]. Our discoveries are consistent with a finding from another laboratory showing that low blood levels of 1,25(OH)_2_D during hospital admission are associated with sequential organ failure (SOFA) in critically ill patients [8].

These previous findings on the association of 1,25(OH)_2_D deficiency with sepsis mortality are in agreement with recent discoveries that 1,25(OH)_2_D is important for maintaining the normal biological functions which are severely perturbed in sepsis patients [9–13]. These exciting discoveries have led to a growing interest in evaluating the potential of vitamin D supplementation for the treatment of sepsis. Accordingly, several clinical trials have been conducted to determine the effects of supplementation of native vitamin D or 1,25(OH)_2_D on disease outcome. However, so far there is no evidence to support that supplementation of native vitamin D can improve disease outcome [14]. Additionally, a clinical trial was conducted to evaluate a single intravenous dose of 1,25(OH)_2_D (2 μg) in patients with severe sepsis or septic shock [15]. The data showed that the 1,25(OH)_2_D supplementation, when compared to placebo control, increased leukocyte mRNA expressions of cathelicidin and interleukin-10 24 h after the treatments. No other significant changes were observed, such as blood cytokine levels (interleukin-10 [IL-10], interleukin-6 [IL-6], tumor necrosis factor-α [TNF-α], interleukin-1β [IL-1β], and interleukin-2 [IL-2]), urinary kidney injury markers, and clinical outcomes. These negative data coupled with the known immune regulatory and antimicrobial functions of 1,25(OH)_2_D raise the possibility that the disorders leading to the 1,25(OH)_2_D deficiency also contribute to mortality among sepsis patients.

Accordingly, using blood samples of the same sepsis patient cohort from our previous study [7] and animals induced for sepsis, this study investigated the mechanisms underlying the sepsis-associated 1,25(OH)_2_D deficiency. We reasoned that such study should identify the mechanisms that can be potentially targeted for the correction of 1,25(OH)_2_D deficiency and for the improvement of survival among sepsis patients.

## Methods

### Study participants

Sepsis patient samples were the stored plasma samples from subjects previously enrolled at our multi-center observational study from January 2005 through June 2006 [16]. In addition, 20 de-identified age- and gender-matched healthy control blood samples were obtained from BioreclamationIVT (New York, USA). The healthy donors had no significant kidney, liver, or cardiovascular diseases. Study participants were enrolled with written informed consent. This human study was approved by the Loma Linda University Institutional Review Board for Human Research (IRB).

### Animals

Six to eight weeks old C57BL/6 mice were used in this study and were purchased from the Jackson Laboratory. The animal study was approved by Loma Linda Institutional Animal Care and Use Committee (IACUC).

### Method of anesthesia

Animals were anesthetized if necessary by isoflurane inhalation at 3.5–4.5% during induction phase and at 3% during maintenance phase.

### Method of euthanasia

At the end of experiments, mice were euthanized in a high CO_2_ environment inside a sealed container that allowed for the mice to be observed during euthanasia. The number of mice euthanized at one time did not exceed the recommended number for the size of the container. Additionally, cervical dislocation was used as the second method to ensure that the animals did not revive. This method was consistent with the recommendations of the Panel on Euthanasia of the American Veterinary Medical Association.

### Mouse sepsis model

Sepsis was induced in C57BL/6 mice with the cecal ligation and puncture approach, as previously described [17, 18]. Livers and kidneys were harvested at euthanasia for RNA isolation. Animal studies were approved by the Loma Linda University Institutional Animal Care and Use Committee.

### Measurement of human biomarkers

Human blood samples were stored at -80 °C until assay without any freeze and thaw cycle. 1,25(OH)_2_D concentration was determined with a radioimmunoassay by the Heartland Assay Laboratory (Ames, Iowa). Calcium colorimetric was purchased from BioVision Inc. (Milpitas, CA, USA). Intact PTH was measured at the Loma Linda Medical Center as part of routine laboratory tests. Growth hormone (GH) and IGF-1 were examined by enzyme-linked immunoassay (ELISA) using respective commercial kits purchased from R&D Systems (Minneapolis, MN). Creatinine was measured by ELISA using a commercial kit from either Cayman Chemical (Ann Arbor, MI) or R&D Systems (Minneapolis, MN).

### Measurement of murine biomarkers

Mouse PTH ELISA kit and cyclic AMP ELISA kit were purchased from LifeSpan BioSciences (Seattle, WA) and Enzo Lifesciences (Farmingdale, NY), respectively. Calcium colorimetric was purchased from BioVision Inc. (Milpitas, CA, USA). IL-6 was determined using a mouse cytometric bead array kit (BD Biosciences, San Diego). GH and IGF-1 ELISA kits were purchased from R&D Systems® (Minneapolis, MN, USA). 1,25(OH)_2_D EIA kit was purchased from immunodiagnostic systems® (Fountain Hills, AZ, USA). Creatinine was examined by ELISA using a commercial kit from R&D Systems (Minneapolis, MN). All assays were performed according to the manufacturer’s instructions. All experiments were performed using 5 mice per group with duplicates.

### RNA isolation and real-time polymerase chain reaction

Total RNA was isolated using an RNeasy Micro Kit® (Qiagen, Valencia, CA) according to the manufacturer’s instructions. First strand cDNA synthesis was performed using the SuperScript® III Reverse Transcriptase (Life Technologies, Grand Island, NY). Quantities of specific mRNAs were determined by real-time PCR and normalized against the house-keeping gene, GAPDH mRNA, using specific primers. A list of primers used in this study is shown in Table 1.

**Table 1:** PCR Primers Used in this Study

Genes	Forward primers	Reverse primers
GHR	5′-AGGTCTCAGGTATGGATCTTTGTCA-3′	5′-GCCAAGAGTAGCTGGTGTAGCCT-3′
IGF-1	5′-GCTTGCTCACCTTCACCAGC-3′	5′-AATGTACTTCCTTCTGAGTCT-3′
IGFBP3	5′-GACGACGTACATTGCCTCAG-3’	5′-GTCTTTTGTGCAAAATAAGGCATA-3’
Socs3	5′-GCTCCAAAAGCGAGTACCAGC-3’	5′-AGTAGAATCCGCTCTCCTGCAG-3’
CYP27B1	5′-ACCCGACACGGAGACCTTC-3’	5′-ATGGTCAACAGCGTGGACAC-3’
GAPDH	5′-AATCCCATCACCATCTTCCA-3’	5′-TGGACTCCACGACGTACTCA-3’

### Statistical analysis

Data were presented as mean ± standard deviation. Shapiro-Wilks test was used to test the normality. t-test or Mann-Whitney U test was used to test the difference of continuous variables. Fisher’s exact test was used for the analysis of binomial variables. The two-tailed test was used for all variables. Correlation between continuous variables was performed using Spearman correlation analysis. *P* < 0.05 was considered statistically significant. All the statistical analyses were performed using GraphPad Prism® (GraphPad, San Diego, CA, USA) or SPSS Statistics 22.

## Results

### Sepsis patients in general displayed suppressed blood 1,25(OH)_2_D levels which were associated with disorders of the mechanisms that regulate 25-hydroxyvitamin D 1α-hydroxylase

In this current study, we obtained blood samples from healthy control subjects to compare with the total population of sepsis patients (survivors and non-survivors). Table 2 shows baseline characteristics of 20 healthy control subjects and 79 sepsis patients. Age and gender were similar between the two groups. However, ethnicity varied. Mortality of the sepsis patients was 11.4%.

**Table 2:** Characteristics of Sepsis Patients and Health Controls

Categories		Health Controls (*n* = 20)	Sepsis Patients (*n* = 79)
Age, years		61.8 ± 3.7	59.1 ± 2.0
Male: Female ratio		9:11	38:41
Ethnicity	Caucasian, n(%)	6 (30)	50 (63.3)
	Hispanic, n(%)	7 (35)	14 (17.7)
	Asian, n(%)	0 (0)	10 (12.7)
	Black, n(%)	7 (35)	5 (6.3)
Comorbidities			
None, n(%)		20 (100)	18 (22.8)
Cardiovascular, n(%)		0 (0)	32 (40.5)
Respiratory, n(%)		0 (0)	23 (29.1)
Diabetes, n(%)		0 (0)	16 (20.3)
Chronic kidney disease, n(%)		0 (0)	6 (7.6)
Infection focus			
Respiratory tract infection, n(%)		N/A	28 (35.4)
Urinary tract infection, n(%)		N/A	26 (32.9)
Intra-abdominal infection, n(%)		N/A	14 (17.7)
Others		N/A	11 (13.9)
Acute Physiology and Chronic Health Evaluation II		N/A	13.5 ± 3.7
30-day mortality, n(%)		N/A	9 (11.4)

**Fig. 1:**
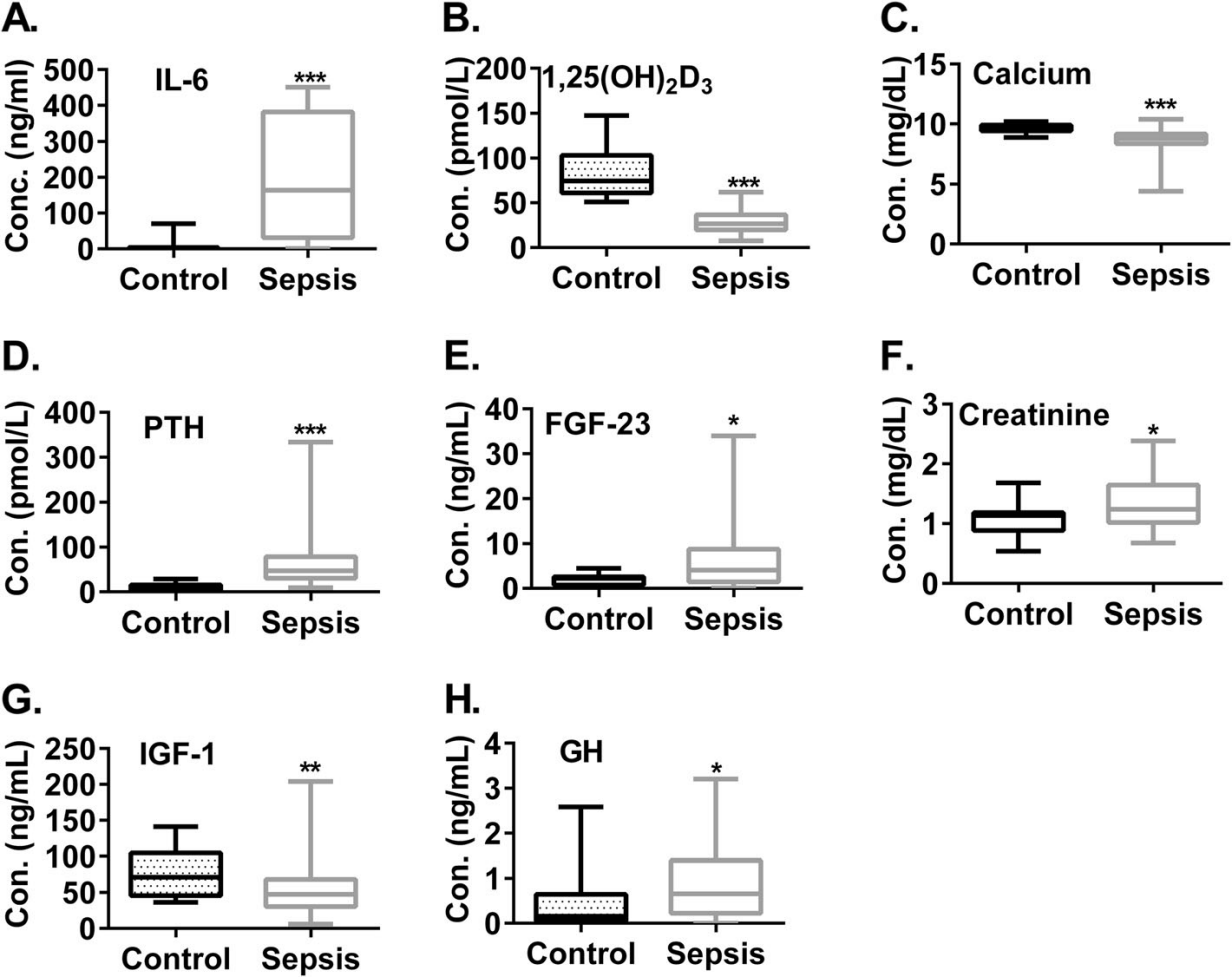
Sepsis patients in general displayed suppressed blood 1,25(OH)_2_D levels which were associated with disorders of the mechanisms that regulate 1α-hydroxylase. Blood samples from 20 age- and gender-matched healthy subjects and 79 sepsis patients were examined for the concentrations of IL-6 (**a**), 1,25(OH)_2_D (**b**), calcium (**c**), PTH (**d**), FGF-23 (**e**)**,** Creatinine (**f**), IGF-1 (**g**), and GH (**h**). Except for the calcium that was measured by colorimetric assay, other molecules were measured by ELISA. *P < 0.05. **P < 0.01, ***P < 0.001. t-test or Mann-Whitney U test

Our data showed that sepsis patients, when compared to healthy control subjects, had significantly increased blood levels of IL-6 (Fig. 1a and Additional file 1: Table S1, 184.2 ± 25.8 vs. 6.2 ± 3.4 ng/mL, *p* < 0.001), a data that supports ongoing inflammation in the sepsis patients. A previous report showed that critical ill patients, when compared to healthy control subjects, had significantly lower blood levels of 1,25(OH)_2_D [19]. Consistent with this previous finding, blood 1,25(OH)_2_D levels in our sepsis patients, when compared to healthy control subjects, were significantly decreased (Fig. 1b and Additional file 1: Table S1, 28.8 ± 1.5 vs. 83.1 ± 6.4 pmol/L, *p* < 0.001). This decreased blood 1,25(OH)_2_D levels in sepsis patients were consistent with significantly decreased blood calcium levels (Fig. 1c and Additional file 1: Table S1, 8.9 ± 0.2 vs. 9.7 ± 0.1 mg/dL, p < 0.001). Collectively, data from our studies and others have demonstrated that 1,25(OH)_2_D deficiency is present in all sepsis patients.

Because the major source of blood 1,25(OH)_2_D is kidney where 1,25(OH)_2_D is converted from 25-hydroxyvitamin D [25(OH)D] by 1α-hydroxylase [25(OH) D is the major vitamin D form in blood] [20], we further evaluated the mechanisms that regulate kidney 1α-hydroxylase. Our data showed that, when compared to healthy control subjects, sepsis patients displayed markedly higher blood levels of PTH (Fig. 1d and Additional file 1: Table S1, 68.2 ± 7.7 vs. 10.6 ± 1.7 pmol/L, p < 0.001), FGF-23 (Fig. 1e and Additional file 1: Table S1, 7.3 ± 2.0 vs. 1.9 ± 0.4 ng/mL, *p* < 0.05), and creatinine (Fig. 1f and Additional file 1: Table S1, 1.5 ± 0.2 vs. 1.0 ± 0.1, p < 0.05), whereas showed significantly decreased blood levels of IGF-1 (Fig. 1g, 55.0 ± 3.7 vs. 77.3 ± 7.4 ng/mL, *p* < 0.01). To support the decreased blood levels of IGF-1 during sepsis, sepsis patients, when compared to healthy control subjects, displayed significantly increased blood levels of growth hormone (GH) (Fig. 1h, 0.98 ± 0.1 vs. 0.49 ± 0.2 ng/mL, p < 0.05) which normally responds to decreased blood levels of IGF-1 [21].

Because we previously reported that, among sepsis patients, non-survivors when compared to survivors had significantly lower blood 1,25(OH)_2_D levels [7], we compared the above measurements between nor-survivors and survivors. Our current data from a different time point of this patient cohort further support the previous finding (Additional file 1: Figure S1 and Table S2, 20.4 ± 3.4 vs. 29.7 ± 1.6 pmol/L, p < 0.05).

In addition, among the sepsis patients, six patients had chronic kidney disease (Table 2). We therefore asked whether the chronic kidney disease might confound our data analysis because kidney failure impairs its 1α-hydroxylase activity and hence blood 1,25(OH)_2_D levels [7]. To address this question, we re-analyzed the data by excluding the data from the six patients who had chronic kidney disease. Our data showed that all the observations were similar to what observed when all patients were included (Additional file 1: Figure S2).

**Fig. 2:**
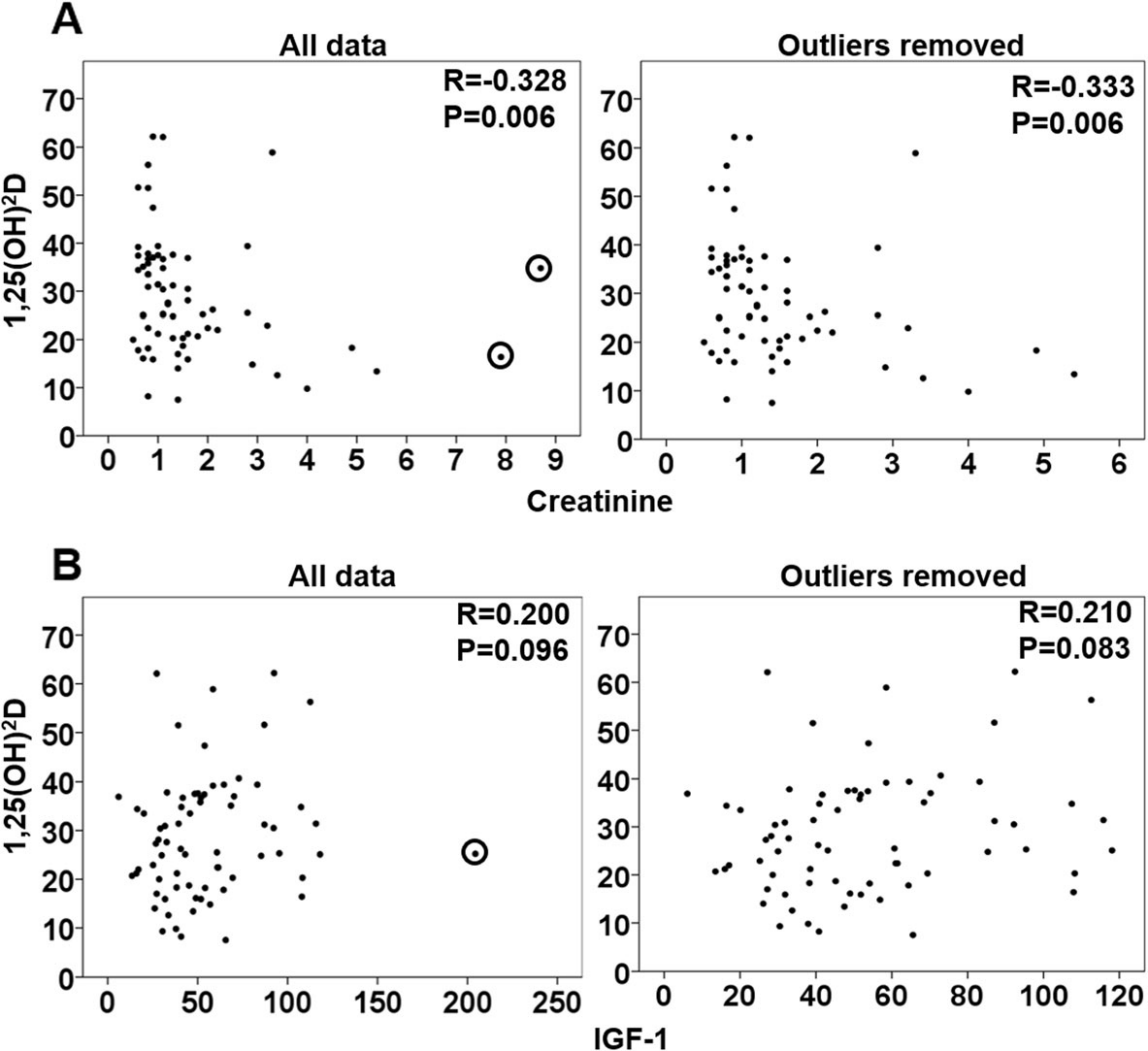
Increased blood creatinine levels and decreased blood IGF-1 levels correlated with suppressed blood 1,25(OH)_2_D levels in sepsis patients. Correlations of blood 1,25(OH)_2_D levels with blood levels of creatinine (**a, left panel**) and IGF-1 (**b, left panel**) in the 79 sepsis patients were analyzed by Spearman correlation analysis. The same analyses were performed after outliers were removed (**a, right panel and b, right pane**l). Circled dots were considered as outliers

Collectively, our data suggest that in sepsis patients multiple disorders negatively affect kidney 1α-hydroxylase and hence blood 1,25(OH)_2_D levels. Such disorders include decreased serum IGF-1 levels, kidney failure as indicated by the increased blood creatinine levels, and increased blood FGF-23 levels. As a result, the suppressed kidney 1α-hydroxylase activity cannot be overcome by the increased blood PTH levels. Based on the above observations, we continued to ask whether the suppressed blood 1,25(OH)_2_D levels correlated with the decreased blood IGF-1 levels and increased blood creatinine levels (kidney failure may directly impair kidney 1α-hydroxylase [22] or indirectly via increased blood FGF-23 levels [23]; therefore, blood creatinine levels were used for this analysis). Using Spearman correlation analysis, our data showed that both blood levels of creatinine (Fig. 2a, R = -0.333, *p* = 0.006) and IGF-1 (Fig. 2b, R = 0.210, *p* = 0.083) moderately correlated with blood 1,25(OH)_2_D levels. These data further support that multiple mechanisms including decreased serum IGF-1 levels and kidney failure/increased blood FGF-23 levels contribute to the suppressed blood 1,25(OH)_2_D levels in sepsis patients.

### Sepsis mice displayed suppressed blood 1,25(OH)_2_D levels which were associated with disorders of the mechanisms that regulate 1α-hydroxylase

**Fig. 3:**
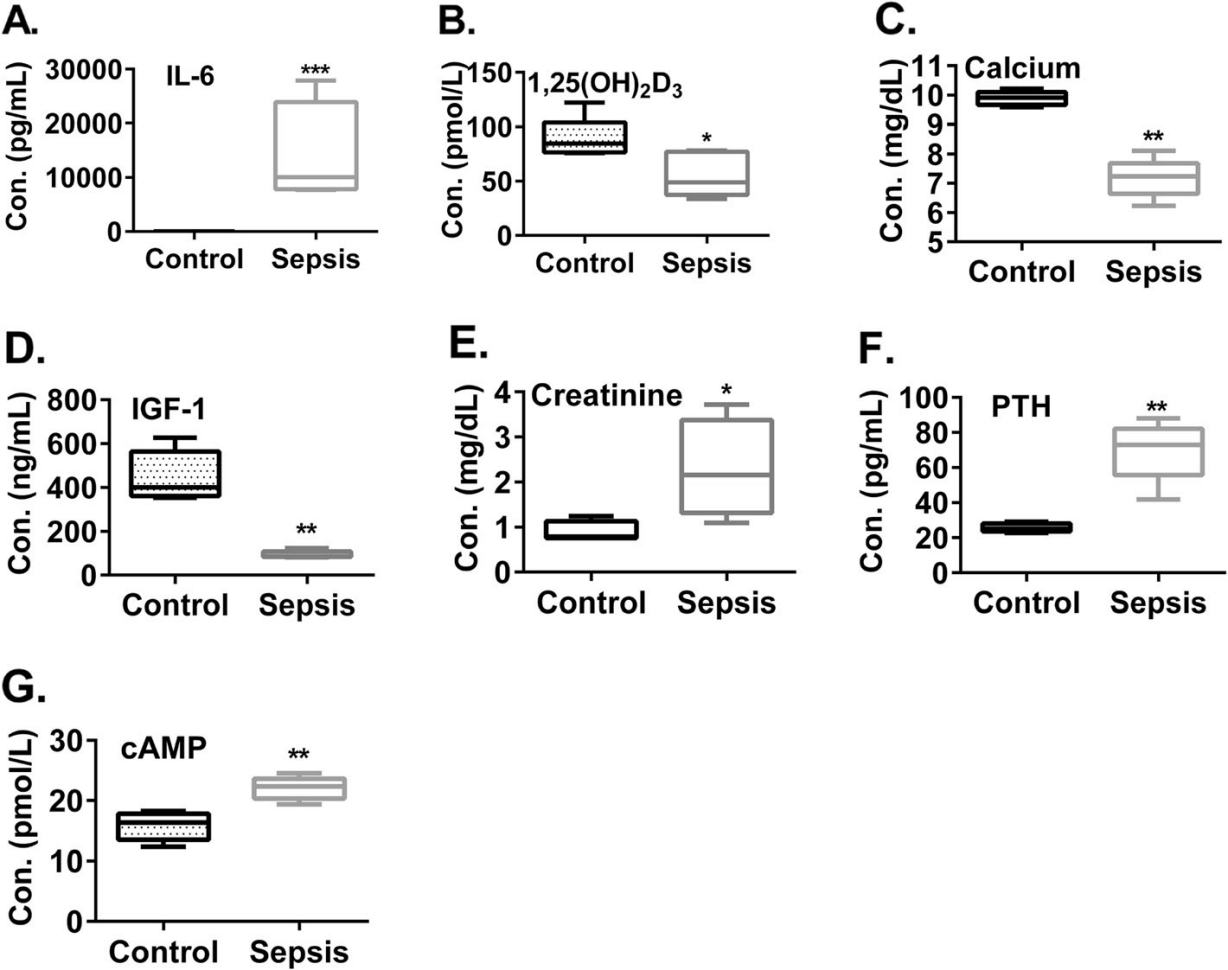
Sepsis mice displayed suppressed blood 1,25(OH)_2_D levels which were associated with disorders of the mechanisms that regulate 1α-hydroxylase. C57BL/6 mice were induced for sepsis by cecal ligation and puncture. Twenty-four hours later, blood samples were collected and examined for the concentrations of IL-6 (**a**), 1,25(OH)_2_D (**b**), calcium (**c**), IGF-1 (**d**), creatinine (**e**), intact PTH (iPTH) (**f**), and cAMP (**g**). *N* = 5. *P < 0.05. t-test or Mann-Whitney U test

To further shed light on our discoveries in human sepsis patients, we decided to extend our human study to mice induced for sepsis. Accordingly, C57BL/6 mice were induced for sepsis by cecal ligation and puncture. Twenty-four hours later, sera were collected for analysis. Our data showed that blood IL-6 levels were significantly increased in sepsis mice when compared to healthy control mice [Fig. 3a and Additional file 1: Table S3, 13,952 ± 4744 pg/mL vs. 1.4 ± 0.0, *p* < 0.05)], consistent with ongoing inflammation in the sepsis mice. Additionally, compared to healthy control mice, sepsis mice also displayed significantly decreased blood 1,25(OH)_2_D levels (Fig. 3b and Additional file 1: Table S3, 55.6 ± 9.1 vs. 89.1 ± 8.5 pmol/mL, p < 0.05), a finding which was consistent with reduced blood calcium levels (Fig. 3c and Additional file 1: Table S3, 7.2 ± 0.3 vs. 9.9 ± 0.1 mg/dL, *p* < 0.01) that is positively regulated by 1,25(OH)_2_D. Also consistent with the findings in human sepsis patients, sepsis mice showed significantly decreased blood IGF-1 levels (Fig. 3d and Additional file 1: Table S3, 93.2 ± 7.5 vs. 449.5 ± 51.0 ng/mL, *p* < 0.01) and increased blood creatinine levels (Fig. 3e and Additional file 1: Table S3, 2.3 ± 0.5 vs. 0.9 ± 0.1 mg/dL, *p* < 0.05). The data suggest that kidney 1α-hydroxylase is negatively affected by multiple mechanisms in the sepsis mice, similar to human sepsis patients. Moreover, sepsis mice had increased blood PTH levels (Fig. 3f and Additional file 1: Table S3, 69.8 ± 7.6 vs. 25.7 ± 1.1 pg/mL, *p* < 0.01), which was in line with increased blood levels of 3′,5′-cyclic adenosine monophosphate (cAMP) (Fig. 3g, and Additional file 1: Table S3. 22.1 ± 0.8 vs.15.8 ± 1.1 pmol/mL, *p* < 0.01) which reflects PTH activity. Hence, also similar to human sepsis patients, sepsis mice displayed suppressed blood 1,25(OH)_2_D levels which cannot be overcome by increased blood PTH levels.

### Suppression of IGF-1 production in liver during sepsis

**Fig. 4:**
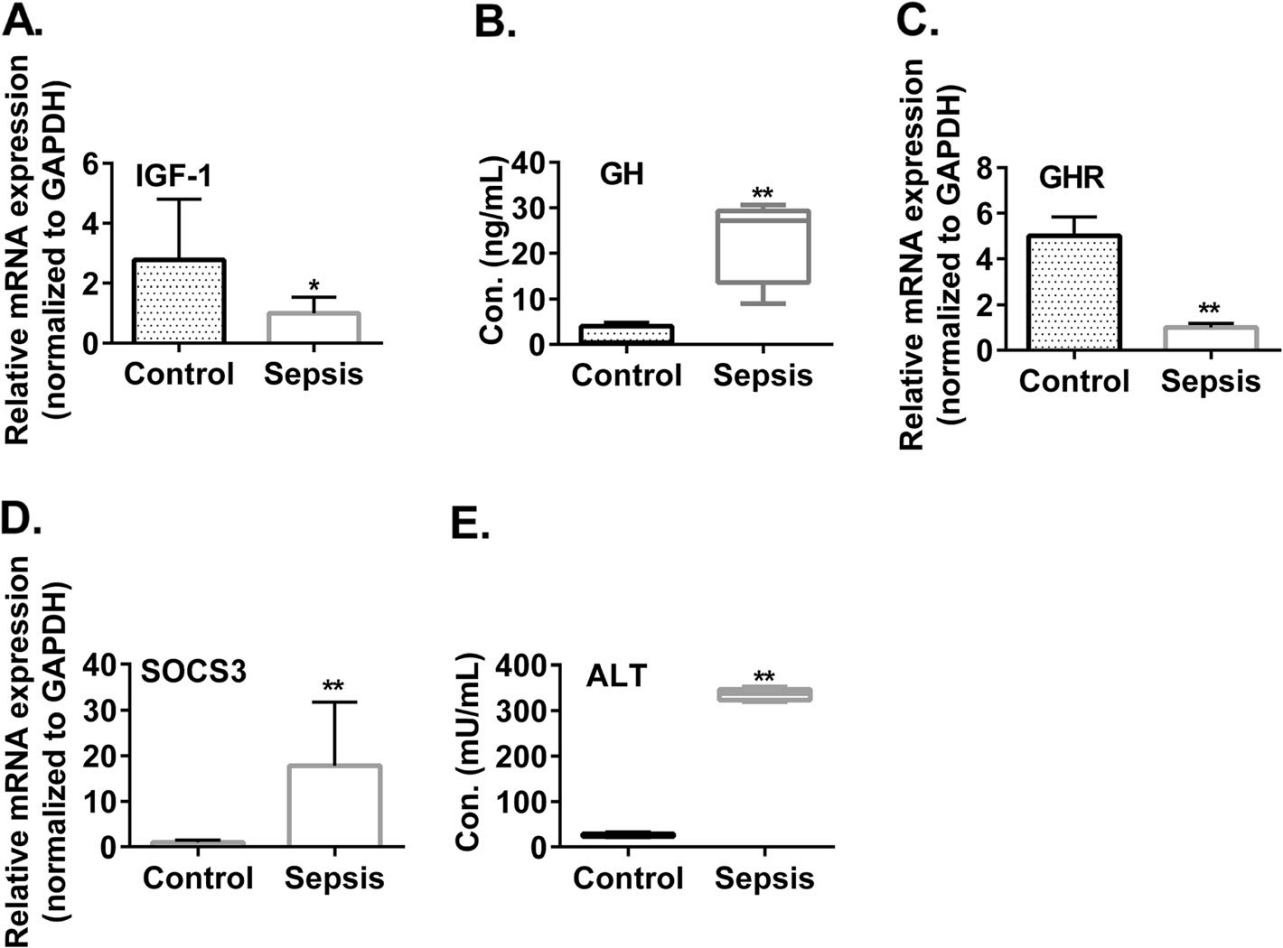
Suppression of IGF-1 production in liver during sepsis. C57BL/6 mice were induced for sepsis by cecal ligation and puncture. Twenty-four hours later, blood samples and liver tissues were collected. The liver tissues were examined by RT-qPCR for the mRNA expressions of IGF-1 (**a**), GHR (**c**), and SOCS3 (**d**). Additionally, the blood samples were examined for the concentrations of growth hormone (GH) (**b**) and ALT (**e**). N = 5. *P < 0.05. t-test or Mann-Whitney U test

Since the increase in blood FGF-23 levels is known to associate with kidney failure [24], we further investigated the mechanisms leading to the sepsis-induced decrease in blood IGF-1 levels. In this regard, it is known that liver is the major tissue where IGF-1 is produced [25]. Our data showed that, indeed, the mRNA expression of IGF-1 was significantly suppressed in the livers of sepsis mice when compared to healthy control mice (Fig. 4a, 1 ± 0.4 vs. 2.4 ± 1.7, p < 0.05). Consistent with the suppressed IGF-1 production, blood levels of GH were significantly increased in sepsis mice (Fig. 4b and Additional file 1: Tables S3, 22.6 ± 4.0 vs. 1.8 ± 1.0 ng/mL, p < 0.01). Therefore, it was evident that the up regulation of GH failed to restore normal production of IGF-1 in sepsis mice, similar to human sepsis patients. We hence studied the pathway that could potentially affect IGF-1 production in liver. Our data showed that the mRNA expression of growth hormone receptor (GHR) was significantly decreased in the livers of sepsis mice when compared to those of healthy control mice (Fig. 4c, 1 ± 0.17 vs. 5.0 ± 0.8, p < 0.01). In addition, the mRNA expression of the suppressor of cytokine signaling 3 (SOCS3), which is a negative regulator of GHR signaling [26], was significantly increased (Fig. 4d, 17.8 ± 8.5 vs. 1.0 ± 0.4, p < 0.01). Moreover, we observed increased blood levels of alanine aminotransferase (ALT) in the sepsis mice, indicating liver failure (Fig. 4e and Additional file 1: Table S3, 334.7 ± 42.1 vs. 23.4 ± 6.2 mU/mL, p < 0.01). Collectively, the above data suggest that both suppressed expression and signaling of GHR and liver failure may contribute to the decreased production of IGF-1 in liver.

## Discussion

Our finding of sepsis-induced 1,25(OH)_2_D deficiency in both human sepsis patients and sepsis mice is clinically significant. Firstly, there is a breakdown of epithelial barrier during sepsis, resulting in local bacterial invasion. In this regard, 1,25(OH)_2_D stimulates the expression of tight junction proteins and hence protects epithelial barrier [9]. Additionally, this barrier-protection function of 1,25(OH)_2_D also helps to prevent vascular leakage [10] which is a serious complication in sepsis. Secondly, 1,25(OH)_2_D stimulates, in immune cells, the production of cathelicidin [11] which acts as an endogenous antimicrobial agent and helps to reduce bacteria burden in sepsis patients. Thirdly, 1,25(OH)_2_D blocks the production of angiopoietin [12] that plays an important role in the pathogenesis during sepsis. Fourthly, 1,25(OH)_2_D suppresses inflammation [9, 13]. However, previous clinical trials of supplementations of native vitamin D and 1,25(OH)_2_D did not produce desired outcomes [14, 15]. For this reason, we reason that the disorders leading to the 1,25(OH)_2_D deficiency also contribute to mortality among sepsis patients.

Under normal conditions, blood 1,25(OH)_2_D levels are controlled by multiple mechanisms including genetic factors [27], blood IGF-1 levels [28, 29], blood FGF-23 levels [27], and blood 25(OH)D levels [29]. It has been shown that blood IGF-1 levels positively [28, 29] and blood FGF-23 levels negatively [27] correlate with blood 1,25(OH)_2_D levels. However, blood 25(OH)D levels do not correlate with blood 1,25(OH)_2_D levels [27, 30]. Blood 1,25(OH)_2_D levels change only when blood 25(OH)D levels change dramatically [30]. In our patient cohort, blood 25(OH)D levels at hospital admission were 21.5 ± 1.1 ng/mL [7] which are defined as vitamin D insufficiency but not deficiency [31]. Although the potential contribution of decreased blood 25(OH)D levels cannot be excluded, based on the foregoing previous findings, we reason that decreased blood IGF-1 levels and increased blood FGF-23 levels are the major contributors to the suppressed blood 1,25(OH)_2_D levels.

In our current study, we found that sepsis patients had increased blood FGF-23 levels (Fig. 1e). In addition to negatively regulating kidney 1α-hydroxylase activity [23, 32, 33], FGF-23 has other important biological functions because it has been shown that the increase in blood FGF-23 levels is associated with all-cause mortality [34]. A recent open-label randomized trial showed that a FGF-23 blocking antibody (i.e. Burosumab) reduced the severity of rickets in patients with X-linked hypophosphataemia. In this regard, X-linked hypophosphataemia is a monogenetic disease that is characterized by FGF-23 overproduction [35]. However, an intriguing finding was that FGF-23 neutralization, although improving chronic kidney disease-associated hyperparathyroidism, increased mortality in a rat model of chronic kidney disease [36]. Therefore, whether blockage of FGF-23 is beneficial to sepsis patients requires further investigation.

Another important finding in our current study is the decreased blood levels of IGF-1 in sepsis patients (Fig. 1g) and sepsis mice (Fig. 3d). In this regard, in addition to its role in stimulating the activity of kidney 1α-hydroxylase [37, 38], IGF-1 can potentially ameliorate kidney and liver failure in sepsis patients [39, 40]. Moreover, IGF-1 has other important biological functions that can be potentially beneficial to sepsis patients, such as suppression of inflammation by enhancing the induction of regulatory T cells and by decreasing bacterial translocation [41–43]; maintenance of normal gonadal hormone production [44]; protection of mitochondrial function; anti-apoptosis; and protection from vascular leakage [45–47].

Accordingly, the cause of sepsis-induced IGF-1 deficiency is of considerable interest. In this regard, sepsis can cause IGF-I deficiency by disrupting the production of GH or by producing a resistance to the action of GH to synthesize IGF-I in the liver which is the major source of circulating IGF-I [46]. We found increased blood GH levels in both sepsis patients (Fig. 1h) and sepsis mice (Fig. 4b). This inability of the elevated GH to bring serum IGF-1 to the normal levels suggests a compromised function of GHR in liver. Indeed, this notion is supported by three findings in the livers of sepsis mice. Firstly, it was shown that in sepsis mice the expression of GHR mRNA in liver was significantly suppressed (Fig. 4c). Secondly, the expression of SOCS3 mRNA in liver was significantly increased (Fig. 4d). In this regard, SOCS3 is a negatively regulator of GHR signaling [26, 48]. We reason that the increased SOCS3 expression in liver is due to proinflammatory cytokines secreted during sepsis (such IL-6 as shown in Fig. 1a and Fig. 3a) because proinflammatory cytokines have been shown to up regulate SOCS3 [26]. Thirdly, there was a liver failure in the sepsis mice (Fig. 4e).

**Fig. 5:**
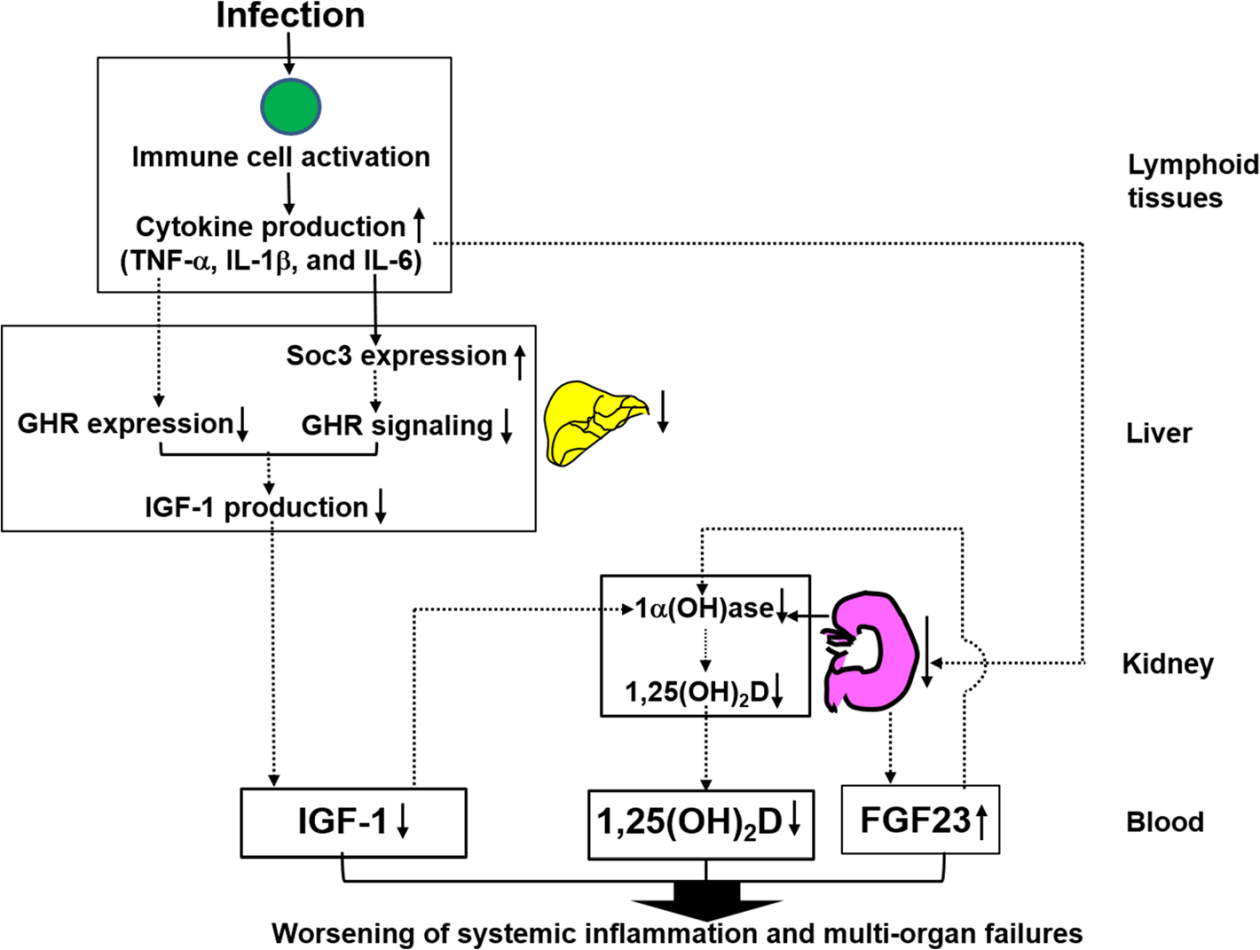
A model for the mechanisms underlying sepsis-induced 1,25(OH)_2_D deficiency. See text for description

Based on our findings, we are proposing a model for the mechanisms underlying sepsis-induced 1,25(OH)_2_D deficiency (Fig. 5). In the model, an infection leads to activation of immune cells followed by secretion of pro-inflammatory cytokines, e.g. TNF-α, IL-1β, and IL-6. These cytokines and liver failure cause inhibition of the expression and signaling of GHR in liver, which leads to reduced production of IGF-1 in liver and decreased IGF-1 levels in blood. In addition, kidney failure increases FGF-23 levels in blood. Consequently, the decreased blood levels of IGF-1, increased blood levels of FGF-23, and kidney failure cause suppressed activity of kidney 1α-hydroxylase, leading to reduced production of 1,25(OH)_2_D in kidney and decreased 1,25(OH)_2_D levels in blood. Collectively, the increased FGF-23 levels, decreased IGF-1 levels, and decreased 1,25(OH)_2_D levels in blood worsen systemic inflammation and multi-organ failures in sepsis patients.

There are several limitations in this study. One is that the blood samples from healthy control subjects were not a part of our previous prospective study. The other limitation is that our study is an association study and not a cause-and-effect result. The third limitation is that, because human creatinine levels measured by the commercial kit all trended high in Fig. 1f, all the data in this figure were divided by 2 from the original data to ensure that creatinine levels in normal healthy control subjects fell into the physiological range. However, this data transformation should not compromise the interpretation in this study because our purpose is to compare normal healthy controls with sepsis patients.

## Conclusions

In this current study, we identified potential mechanisms that lead to the suppressed production of 1,25(OH)_2_D in sepsis patients. Our study suggests that supplementations of native vitamin D or 1,25(OH)_2_D alone may not be sufficient for the treatment of sepsis. Therefore, in order to improve sepsis survival, new therapeutic strategies are needed to target the upstream mechanisms that lead to the suppressed production of 1,25(OH)_2_D. Such upstream mechanisms include decreased production of IGF-1, increased production of FGF-23, and kidney failure.

## Supplementary information

**Additional file 1: Table S1.** Patient sample measurement results. **Table S2.** Measurements between survivors and non-survivors. **Table S3.** Mouse sample measurement results. **Figure S1.** Blood 1,25(OH)_2_D levels were significantly lowers in non-survivors when compared to survivors among sepsis patients. **Figure S2.** Sepsis patients in general displayed suppressed blood 1,25(OH)_2_D levels which were associated with disorders of the mechanisms that regulate **1α**-hydroxylase.

## Data Availability

Materials are readily available and shall be requested via Dr. Xiaolei Tang (xitang@llu.edu or Xiaolei.tang@liu.edu). Materials will be provided under the material transfer policies of the LLU.

## Abbreviations

1,25(OH)_2_D: 25-dihydroxyvitamin D
1α-hydroxylase: 25-hydroxyvitamin D 1α-hydroxylase
cAMP: 3’, 5’-cyclic adenosine monophosphate
FGF-23: Fibroblast growth fact 23
IGF-1: Insulin-like growth factor-1
PTH: Parathyroid hormone
