# Supplementary material for: Mechanistic study of the cause of decreased blood 1,25-Dihydroxyvitamin D in sepsis
Source: BMC Infect Dis. 2019 Dec 2;19:1020. doi: 10.1186/s12879-019-4529-7 (PMC6888965; doi:10.1186/s12879-019-4529-7)
Supplement: Supplementary file 1 — Additional file 1: Table S1. Patient sample measurement results. Table S2. Measurements between survivors and non-survivors. Table S3. Mouse sample measurement results. Figure S1. Blood 1,25(OH)2D levels were significantly lowers in non-survivors when compared to survivors among sepsis patients. Figure S2. Sepsis patients in general displayed suppressed blood 1,25(OH)2D levels which were associated with disorders of the mechanisms that regulate 1α-hydroxylase. [file 12879_2019_4529_MOESM1_ESM.docx]

**Mechanistic Study of the Cause of Decreased Blood 1,25-Dihydroxyvitamin D in Sepsis**

**(Additional file)**

Chih-Huang Li^1,5,6,¶^, Xiaolei Tang^1,2,¶,*^, Samiksha Wasnik^1^, Xiaohua Wang^1,7^, Jintao Zhang^1,8^, Yi Xu^1^, Kin-Hing William Lau^1,4^, H Bryant Nguyen^1,3^, David J Baylink^1^.

^1^Department of Medicine, Division of Regenerative Medicine, Loma Linda University, Loma Linda, California, USA;

^2^Department of Veterinary Biomedical Sciences, College of Veterinary Medicine, Long Island University, USA;

^3^Division of Pulmonary, Critical Care, Hyperbaric and Sleep Medicine, Loma Linda University, Loma Linda, California, USA;

^4^Musculoskeletal Disease Center, Jerry L. Pettis Memorial Veterans Affairs Medical Center, Loma Linda, California, USA;

^5^Department of Emergency Medicine, Chang-Gung Memorial Hospital, Linkou Medical Center, Taoyuan, Taiwan;

^6^Graduate Institute of Clinical Medical Sciences, School of Medicine, Chang-Gung University, Taoyuan, Taiwan;

^7^Division of Infectious Disease, Jinan Infectious Disease Hospital, Shandong University, Jinan, China;

^8^Institute of Medical and Pharmaceutical Sciences, Zhengzhou University, Henan, China.

**Short title:** Mechanism of suppression of blood 1,25-dihydroxyvitamin D in sepsis.

^¶^**These authors contributed equally to this work**

***Correspondence:** Xiaolei Tang, M.D./Ph.D.; Division of Regenerative Medicine; Department of Medicine; Loma Linda University; Loma Linda, California, USA. Department of Veterinary Biomedical Sciences, College of Veterinary Medicine, Long Island University; Brookville, NY 11548. Email: [XITang@llu.edu](mailto:XITang@llu.edu) or [xtang25@outlook.com](mailto:xtang25@outlook.com).

**Additional file 1: Table S1.** Patient sample measurement results

|  | **Health Controls** | **Sepsis Patients** | **P value** |
| --- | --- | --- | --- |
| **IL-6 (ng/mL)** | 6.2 ± 3.4 | 184.2 ± 25.8 | < 0.001 |
| **1,25(OH)_2_D (pmol/L)** | 83.1 ± 6.4 | 28.8 ± 1.5 | < 0.001 |
| **Calcium (mg/dL)** | 9.7 ± 0.1 | 8.9 ± 0.2 | <0.001 |
| **PTH (pmol/L)** | 10.6 ± 1.7 | 68.2 ± 7.7 | < 0.001 |
| **FGF23 (ng/mL)** | 1.9 ± 0.4 | 7.3 ± 2.0 | <0.05 |
| **Creatinine (mg/dL)** | 1.0 ± 0.1 | 1.5 ± 0.2 | <0.05 |
| **IGF-1 (ng/mL)** | 77.3 ± 7.4 | 55.0 ± 3.7 | <0.01 |
| **GH (ng/mL)** | 0.49 ± 0.2 | 0.98 ± 0.1 | <0.05 |

**Table S2.** Measurements between survivors and non-survivors

|  | **Survivors** | **Non-survivors** | **P value** |
| --- | --- | --- | --- |
| **IL-6 (ng/mL)** | 176.0 ± 27.4 | 256.2 ± 73.9 | 0.35 |
| **1,25(OH)_2_D (pmol/L)** | 29.7 ± 1.6 | 20.4 ± 3.4 | < 0.05 |
| **Calcium (mg/dL)** | 9.0 ± 0.1 | 8.3 ± 0.5 | 0.05 |
| **PTH (pmol/L)** | 65.9 ± 8.1 | 85.0 ± 26.4 | 0.42 |
| **FGF23 (ng/mL)** | 5.1 ± 1.5 | 13.7 ± 6.2 | 0.24 |
| **Creatinine (mg/dL)** | 1.8 ± 0.2 | 1.6 ± 2.6 | 0.75 |
| **IGF-1 (ng/mL)** | 56.4 ± 4.0 | 45.2 ± 7.7 | 0.31 |
| **GH (ng/mL)** | 0.9 ± 0.1 | 1.5 ± 3.7 | 0.06 |

**Table S3.** Mouse sample measurement results

|  | **Control** | **Sepsis** | **P value** |
| --- | --- | --- | --- |
| **IL-6 (pg/mL)** | 1.4 ± 0.0 | 13952 ± 4744 | <0.05 |
| **1,25(OH)_2_D (pmol/L)** | 89.1 ± 8.5 | 55.6 ± 9.1 | <0.05 |
| **Calcium (mg/dL)** | 9.9 ± 0.1 | 7.2 ± 0.3 | < 0.01 |
| **IGF-1 (ng/mL)** | 449.5 ± 51.0 | 93.2 ± 7.5 | <0.01 |
| **Creatinine (mg/dL)** | 0.9 ± 0.1 | 2.3 ± 0.5 | <0.05 |
| **PTH (pg/mL)** | 25.7 ± 1.1 | 69.8 ± 7.6 | <0.01 |
| **cAMP (pmol/mL)** | 15.8 ± 1.1 | 22.1 ± 0.8 | <0.01 |
| **GH (ng/mL)** | 1.8 ± 1.0 | 22.6 ± 4.0 | <0.01 |
| **ALT (mU/mL)** | 23.4 ± 6.2 | 334.7 ± 42.1 | <0.01 |


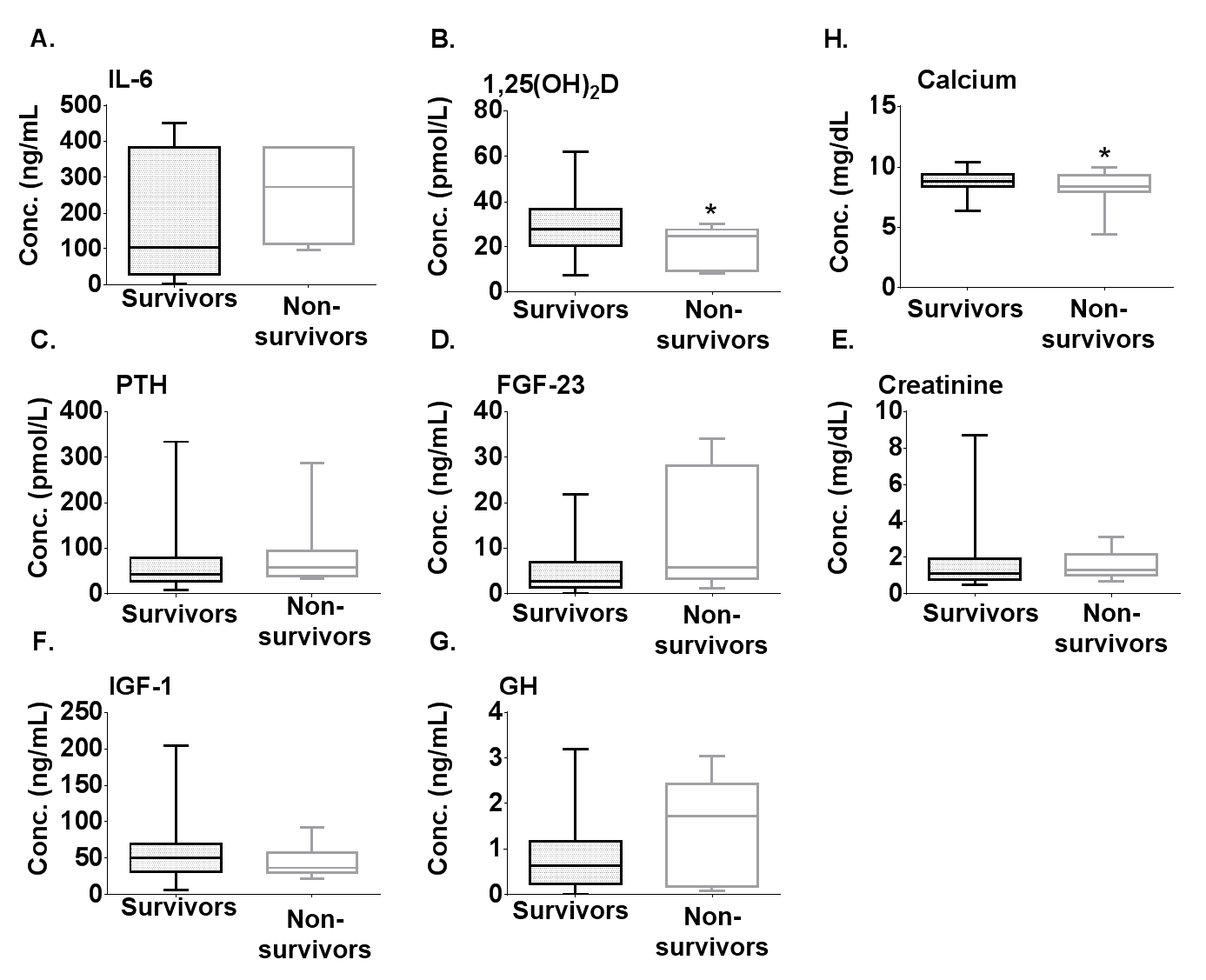


**Figure S1.** **Blood 1,25(OH)_2_D levels were significantly lower in non-survivors when compared to survivors among sepsis patients.** Data of sepsis patients in Figure 1 were further analyzed for the difference between survivors and non-survivors.


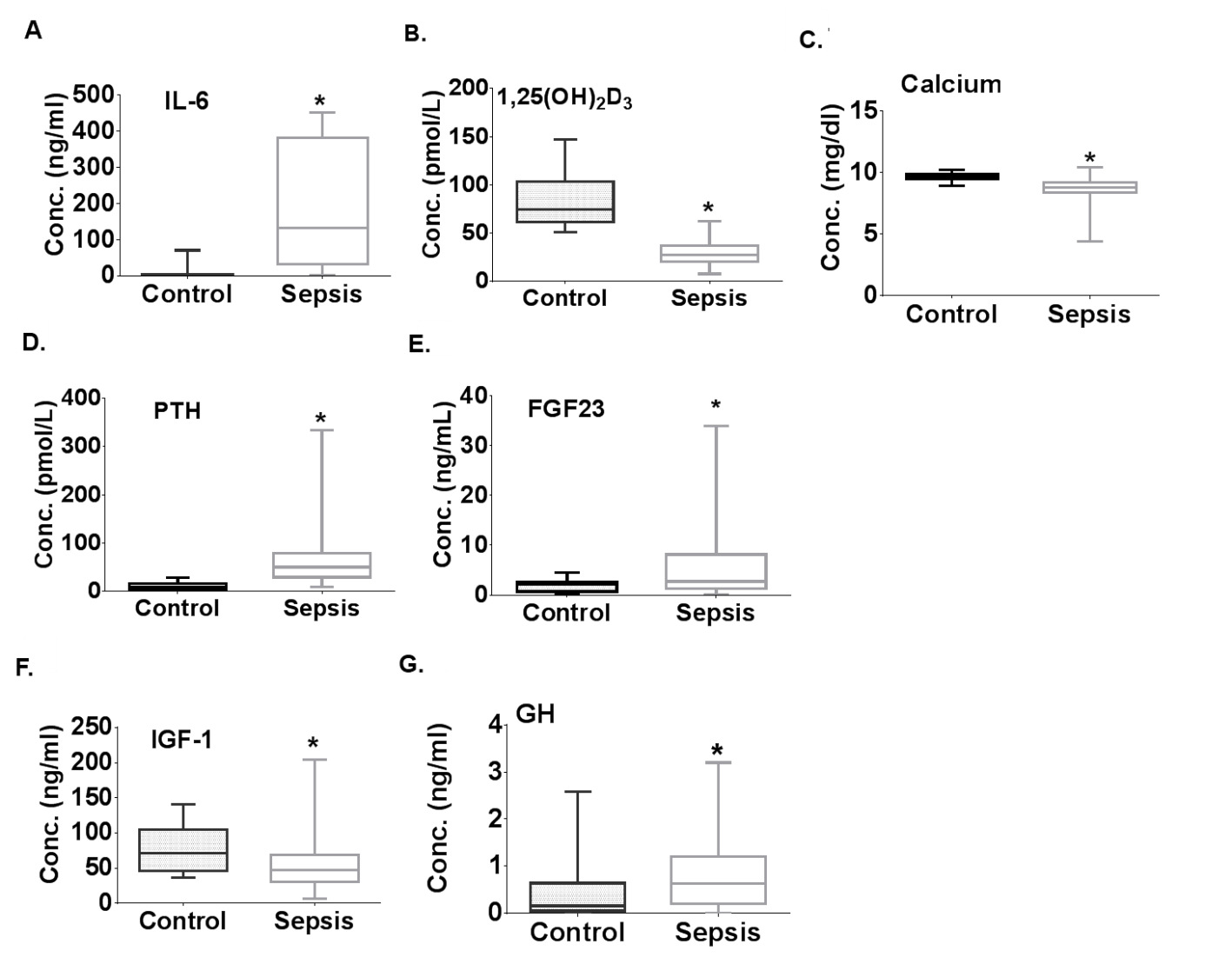


**Figure S2.** **Sepsis patients in general displayed suppressed blood 1,25(OH)_2_D levels which were associated with disorders of the mechanisms that regulate 1α-hydroxylase.** Six sepsis patients who had chronic kidney disease (see Table 2) were excluded from analysis. Also see Figure 1 for complete data analysis.
